# *Noggin* Overexpression Impairs the Development of Muscles, Tendons, and Aponeurosis in Soft Palates by Disrupting BMP-Smad and Shh-Gli1 Signaling

**DOI:** 10.3389/fcell.2021.711334

**Published:** 2021-09-07

**Authors:** Jiamin Deng, Shangqi Wang, Nan Li, Xiaoyan Chen, Biying Wang, Han Liu, Lei Zhu, Wei Cong, Jing Xiao, Chao Liu

**Affiliations:** ^1^Department of Oral Pathology, School of Stomatology, Dalian Medical University, Dalian, China; ^2^Dalian Key Laboratory of Basic Research in Oral Medicine, School of Stomatology, Dalian Medical University, Dalian, China; ^3^Academician Laboratory of Immune and Oral Development and Regeneration, Dalian Medical University, Dalian, China

**Keywords:** palatogenesis, cleft soft palate, BMP signaling, aponeurosis, myogenesis

## Abstract

The roles of bone morphogenetic protein (BMP) signaling in palatogenesis were well documented in the developing hard palate; however, little is known about how BMP signaling regulates the development of soft palate. In this study, we overexpressed *Noggin* transgene via *Osr2-cre*^KI^ allele to suppress BMP signaling in the developing soft palate. We found that BMP-Smad signaling was detected in the palatal muscles and surrounding mesenchyme. When BMP-Smad signaling was suppressed by the overexpressed *Noggin*, the soft palatal shelves were reduced in size with the hypoplastic muscles and the extroversive hypophosphatasia (HPP). The downregulated cell proliferation and survival in the *Osr2-cre*^KI^*;pMes-Noggin* soft palates were suggested to result from the repressed *Shh* transcription and Gli1 activity, implicating that the BMP-Shh-Gli1 network played a similar role in soft palate development as in the hard palate. The downregulated Sox9, *Tenascin-C* (*TnC*), and *Col1* expression in *Osr2-cre*^KI^*;pMes-Noggin* soft palate indicated the impaired differentiation of the aponeurosis and tendons, which was suggested to result in the hypoplasia of palatal muscles. Intriguingly, in the *Myf5-cre*^KI^*;pMes-Noggin* and the *Myf5-cre*^KI^*;Rosa26R-DTA* soft palates, the hypoplastic or abrogated muscles affected little the fusion of soft palate. Although the *Scx*, *Tnc*, and *Co1* transcription was significantly repressed in the tenogenic mesenchyme of the *Myf5-cre*^KI^*;pMes-Noggin* soft palate, the Sox9 expression, and the *Tnc* and *Col1* transcription in aponeurosis mesenchyme were almost unaffected. It implicated that the fusion of soft palate was controlled by the mesenchymal clues at the tensor veli palatini (TVP) and levator veli palatini (LVP) levels, but by the myogenic components at the palatopharyngeus (PLP) level.

## Introduction

The secondary palate of mammals is composed of the hard palate occupying the anterior two-thirds of the entire palate and the soft palates in the posterior one-third of palate (Bush and Jiang, 2012). The developing hard palate is constituted by the covering epithelium that originated from surface ectoderm and the mesenchyme derived from cranial neural crest (Bush and Jiang, 2012; Li et al., 2017). In contrast, the soft palate contains not only the covering epithelium and neural crest-derived mesenchyme differentiating into tendons and other irregular connective tissues (Nassari et al., 2017), but also the myoblasts that originated from the mesoderm-derived branchiomeric mesenchyme (Li et al., 2019). There are five muscles in the human soft palate: tensor veli palatini (TVP), levator veli palatini (LVP), palatoglossus (PLG), palatopharyngeus (PLP), and musculus uvulae. Except musculus uvulae, the other four pairs of muscles attach to the palatine aponeurosis, a fan-like fibrous structure connecting the posterior board of the hard palate (Grimaldi et al., 2015; Li et al., 2019). Despite the absence of musculus uvulae, the anatomy of soft palate in mouse is similar to that in humans (Grimaldi et al., 2015; Li et al., 2019).

The molecular regulatory networks in the anterior and posterior palatal shelves were distinguishable from each other and have been well studied (Li et al., 2017). However, the molecular mechanisms involved in soft palate development were still poorly understood, which could be attributed to the limited animal models recapitulating the cleft soft palate in humans (Li et al., 2019). Additionally, in most previous studies, the posterior palate referred to the most posterior margin of the hard palate, which overlapped the presumptive TVP level, but ignored the LVP and PLP levels of soft palate (Li et al., 2017). Thus, not only the molecular mechanisms controlling the connective tissue formation and myogenesis in soft palate, but also the association of palatal myogenesis and tenogenesis with the morphogenesis of soft palate remains unknown.

Recently, Wnt, FGF, and Hh signaling pathways were reported to be activated in the mesenchyme surrounding the myofibers in the developing soft palate, implicating that these signaling pathways were also critical in the tenogenesis in soft palate (Sugii et al., 2017; Janečková et al., 2019). A previous study found that in the developing mouse soft palate, the epithelial TGFβ signaling repressed *Dkk1* and *Dkk4* transcription in palatal epithelium to maintain the canonical Wnt signaling in the mesenchyme of soft palate (Iwata et al., 2014). Inactivation of *Tgfbr2* in epithelium disrupted the muscle patterning in soft palate by suppressing mesenchymal canonical Wnt signaling (Iwata et al., 2014). Since the tendons in soft palate predominantly originated from mesenchyme (Nassari et al., 2017), this report implied that the epithelial TGFβ signaling controlled muscle patterning by regulating tendon development (Huang et al., 2013).

Previous studies demonstrated that bone morphogenetic proteins (BMPs) play critical roles during palatogenesis. In the developing mouse palates, *Bmp2* and *Bmp4* were expressed in the anterior palate at E13.5 (Zhang et al., 2002) and activated Bmpr1a in the anterior palatal mesenchyme (Lu et al., 2011). Bmp4 and its downstream target, Msx1, maintained *Shh* transcription in the anterior palatal epithelium, which was required for the cell proliferation in the underlying mesenchyme (Zhang et al., 2002). Inactivation of *Bmpr1a* with *Wnt1-cre* or *Osr2-cre* suppressed cell proliferation in the anterior palate (Baek et al., 2011; Lu et al., 2011). From E14.5 to E16.5, *Bmpr1a* was activated in the medial mesenchyme of the posterior palates (Lu et al., 2011), which coincided with the phosphorylated Smad1/5/8 domain (He et al., 2010). Combined with the compromised osteogenesis in the *Osr2-cre;Smad4^*f/f*^* mouse palatine (Parada et al., 2013), these results strongly suggested the indispensable role of BMP-Smad signaling in the osteogenesis of the presumptive palatine. Furthermore, our latest study showed that the suppression on BMP-Smad signaling by overexpressing *Noggin* impaired the osteogenesis of the hard palate in mice by inhibiting osteogenic differentiation in the palatal mesenchyme (Li et al., 2021).

The latest study reported that *Bmp3* and *Bmp7* were activated in the most posterior part of palate at E13.5 (Fu et al., 2017), which suggested an involvement of BMP-Smad signaling in the development of soft palate. To examine whether the mesenchymal BMP signaling contributes to the development of soft palate, we investigate the development of the soft palate in *Osr2-cre*^KI^*;pMes-Noggin* mouse embryos. Since the *Osr2-cre*^KI^*;pMes-Noggin* mouse embryos suffer from a complete cleft palate, to exclude the possibility that the cleft in the soft palate was secondary to the cleft in the hard palate, we focused on the primary defects in the cell survival, connective tissue formation, and myogenesis in the soft palate of *Osr2-cre*^KI^*;pMes-Noggin* mouse embryos.

## Materials and Methods

### Mouse Lines

All the *Osr2-cre*^KI^ (Chen et al., 2009), *pMes-Noggin* (Xiong et al., 2009), *Myf5-cre* (Stock No. 007893), *Rosa26R-mT/mG* (Stock No. 007676), and *Rosa26R-DTA* (Stock No. 009669) mice used in this study were bred in the Specific Pathogenic Free System of the Institute of Genome Engineered Animal Models for Human Diseases at Dalian Medical University by strictly following the Guide for the Care and Use of Laboratory Animals of Dalian Medical University. Genotyping were performed as the previous description (Chen et al., 2009; Xiong et al., 2009) or following Jackson Lab’s instructions. To get the timed pregnant mice, mating of the female mice to male mice were started from 8:00 p.m. until 8:00 a.m. The morning detection vaginal plug was recorded as E0.5. The timed pregnant female mice were sacrificed by cervical dislocation after CO_2_ anesthesia to collect the embryos.

### Cryostat Section

The E16.5 *Osr2-cre*^KI^*;Rosa26R-mT/mG* mouse heads were fixed with a mixture containing 4% paraformaldehyde (PFA) and 15% sucrose overnight at 4°C. Then, the heads were dehydrated in 30% sucrose and embedded in optimum cutting temperature (O.C.T.) compound (Tissue-Tek, Sakura^®^ Finetek, VWR, Torrance, CA, United States) for 10-μm section. Immediately after sectioning, the sections were observed, and images were taken by the Olympus DP72 microscope (Olympus, Tokyo, Japan) as previously described (Tian et al., 2021).

### Paraffin Section and Staining

The embryonic mouse heads at the desired stages were collected in ice-cold phosphate-buffered saline (PBS) and fixed in ice-cold 4% PFA overnight. The fixed samples were dehydrated with gradient alcohols for paraffin embedding. The embedded samples were sectioned crossly at a thickness of 10 μm for Masson staining as previously described (Li et al., 2021).

### Immunohistochemistry and Immunohistofluorescence

Immunohistochemistry staining was performed on the paraffin sections of 10-μm thickness. The primary antibodies used for immunohistochemistry were anti-myosin (ZM0196; Zhongshan Golden Bridge, Beijing, China), anti-phospho-Smad1/5/8 (13820S; 1:200, Cell Signaling Technology, Danvers, MA, United States), and anti-Sox9 (ab185966; Abcam, Cambridge, MA, United States). The horseradish peroxidase (HRP)-conjugated anti-rabbit/mouse IgG and the DAB substrate kit purchased from MXB Biotechnologies Inc. (Fujian, China) were used as the secondary antibody and color development reagent, respectively, as the manufacturer instructed. Methyl green or hematoxylin was used for counter-staining.

For Gli1 immunohistofluorescence, the paraffin sections were incubated with the rabbit IgG (514675; ZEn Bio Inc., Chengdu, China) after re-hydration in the gradient ethanol solutions and washing in PBS. The CY3-conjugated goat anti-rabbit IgG (S0011; Affinity, Cincinnati, OH, United States) was applied as the secondary antibody. The Olympus DP72 microscope was used for fluorescence observation and image collection.

### Cell Proliferation and Apoptosis Assay

To assess the cell proliferation, the immunohistostaining with antibody against Ki67 (511390; ZEN Bio Inc., China) was performed on the 10-μm-thick paraffin sections. The HRP-conjugated anti-rabbit/mouse IgG (MXB Biotechnologies Inc., Fujian, China) was used as the secondary antibody. The color was developed with the DAB substrate kit (MXB Biotechnologies Inc., Fujian, China), following the manufacturer’s instruction. Hematoxylin was used for counter-staining. The TUNEL assay was also performed on 10-μm-thick paraffin sections with the *In Situ* Cell Death Detection Kit, POD (11684817910, Roche Diagnostics Corporation, Indianapolis). The procedure for apoptosis detection followed the manufacturer’s instructions. The sections were counter-stained with DAPI and observed by the Olympus DP72 microscope. The percentages of cell proliferation were determined by the numbers of Ki67-positive nuclear to the numbers of the total nuclei. The densities of cell apoptosis were calculated by TUNEL fluorescence signal in a fixed area. To evaluate whether the differences in cell proliferation and death were significant, Student’s *t*-tests were used to evaluate the pairs of the WT and *Osr2-cre*^KI^*;pMes-Noggin* soft palates. The results were presented as mean ± SD of at least three pairs of samples. When the *p*-value was less than 0.05, the difference was considered to be statistically significant.

### *In situ* Hybridization

Mouse embryonic heads of desired stages were dissected in diethyl pyrocarbonate (DEPC)-treated Dulbecco’s PBS and fixed with 4% PFA in 0.1% DEPC-treated PBS at 4°C overnight. For section *in situ* hybridization (ISH), the fixed heads were dehydrated in a serial gradient of ethanol solutions followed by paraffin embedding and then prepared as the consecutive sections with 10-μm thickness. For whole-mount ISH, the fixed heads were dehydrated in the gradient methanol. The preparation of the sense and anti-sense RNA probes was transcribed from the plasmids containing the mouse *Shh*, *Scx*, *Tenascin-C* (*Tnc*), and *Col1a1* cDNAs as previously described (Wu et al., 2015; Zhu et al., 2017) by using an RNA Labeling Kit (Roche, Indianapolis, IN, United States). The anti-DIG antibody conjugated with AP and the BM Purple (Roche, Indianapolis, IN, United States) were utilized for probe detection and color development. All the sections were counter-stained by nuclear fast red.

## Results

### The Hypoplastic Muscles and Extraversive Hypophosphatasia in *Osr2-cre*^KI^*;pMes-Noggin* Soft Palates

**FIGURE 1:**
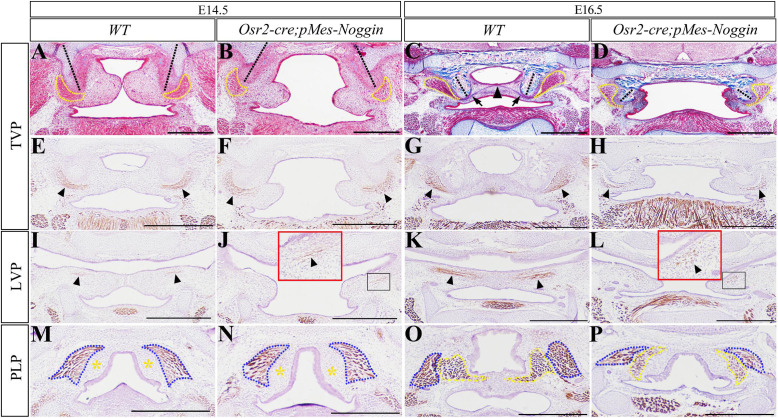
The morphology of HPP and muscles in the *Osr2-cre*^KI^*;pMes-Noggin* soft palates. **(A–D)** Masson staining showed the E14.5 WT soft palate **(A)**, the E14.5 *Osr2-cre*^KI^*;pMes-Noggin* soft palate **(B)**, the E16.5 WT soft palate **(C)**, and the E16.5 *Osr2-cre*^KI^*;pMes-Noggin* soft palate **(D)**. The dashed yellow lines outline the TVPs; the black arrowheads indicate aponeurosis; the black arrows delineate TVP tendons; and the dashed black lines stand for HPP orientation. **(E–H)** The myosin staining showed the TVP myofibers of the E14.5 WT soft palate **(E)**, the E14.5 *Osr2-cre*^KI^*;pMes-Noggin* soft palate **(F)**, the E16.5 WT soft palate **(G)**, and the E16.5 *Osr2-cre*^KI^*;pMes-Noggin* soft palate **(H)**. Black arrows indicate the TVP myofibers. **(I–L)** The immunostaining of myosin showed the LVP myofibers of the E14.5 WT soft palate **(I)**, the E14.5 *Osr2-cre*^KI^*;pMes-Noggin* soft palate **(J)**, the E16.5 WT soft palate **(K)**, and the E16.5 *Osr2-cre*^KI^*;pMes-Noggin* soft palate **(L)**. Black arrows point to the LVP myofibers; red boxes in **(J,L)** are the magnified images in the black boxes. **(M–P)** The myosin staining showed the SPC and PLP myofibers of the E14.5 WT soft palate **(M)**, the E14.5 *Osr2-cre*^KI^*;pMes-Noggin* soft palate **(N)**, the E16.5 WT soft palate **(O)**, and the E16.5 *Osr2-cre*^KI^*;pMes-Noggin* soft palate **(P)**. Blue dotted lines outline the SPC; yellow dotted lines circle the PLP; the yellow asterisks delineate the presumptive PLP areas. TVP, tensor veli palatini; LVP, levator veli palatini; PLP, palatopharyngeus; SPC, superior pharyngeal constrictor; HPP, hypophosphatasia. Scale bars are 200 μm.

The E16.5 *Osr2-cre*^KI^*;Rosa26R-mT/mG* mouse showed green fluorescence in the aponeurosis, the tendon of TVP, and the inferior part of the hamulus of the medial pterygoid palate (Supplementary Figure 1A), but was excluded from the epithelium and the myofibers of TVP, LVP, and PLP (Supplementary Figures 1A–C). Thus, the *Osr2-cre*^KI^ allele could activate *pMes-Noggin* transgene in the mesenchyme of soft palate. In the WT soft palate, the horizontally growing shelves began to contact the TVP level at E14.5 (Figure 1A, and were integrated into a floor containing an aponeurosis at E16.5, Figure 1C). While the shelves in the E14.5 and E16.5 *Osr2-cre*^KI^*;pMes-Noggin* soft palates were obviously smaller and separated from each other, they grew horizontally (Figures 1B,D). It is worth noticing that the orientation of hypophosphatasias was intraversive in the WT soft palates (Figures 1A,C), but extraversive in the *Osr2-cre*^KI^*;pMes-Noggin* soft palates (Figures 1B,D). In addition, both the Masson and Myosin staining indicated that the TVPs in the *Osr2-cre*^KI^*;pMes-Noggin* soft palate were mildly smaller than the WT TVPs at E14.5 (Figures 1A,B,E,F), but reduced dramatically in size at E16.5 (Figures 1C,D,G,H). Myosin staining also showed that when the E14.5 WT LVP myofibers became evident (Figure 1I), only a few LVP myofibers sparsely distributed in the *Osr2-cre*^KI^*;pMes-Noggin* soft palate (Figure 1J). More severely, in contrast to the elongated and denser LVP myofibers at the E16.5 WT soft palate (Figure 1K), the E16.5 *Osr2-cre*^KI^*;pMes-Noggin* LVP myofibers became even looser and shorter than the E14.5 counterparts (Figure 1L). Similarly, although both the E14.5 WT and *Osr2-cre*^KI^*;pMes-Noggin* soft palates showed no difference in superior pharyngeal constrictors (SPCs) and devoid of PLPs (Figures 1M,N), the SPCs and PLPs in the E16.5 *Osr2-cre*^KI^*;pMes-Noggin* soft palate exhibited smaller sizes and the less myofibers than the WT controls (Figures 1O,P). These results suggested that the *Osr2-cre*^KI^*;pMes-Noggin* palatal muscles underwent hypoplasia.

### Suppressed BMP-Smad Signaling and Disrupted Shh-Gli1 Signaling in the *Osr2-cre*^KI^*;pMes-Noggin* Soft Palates

**FIGURE 2:**
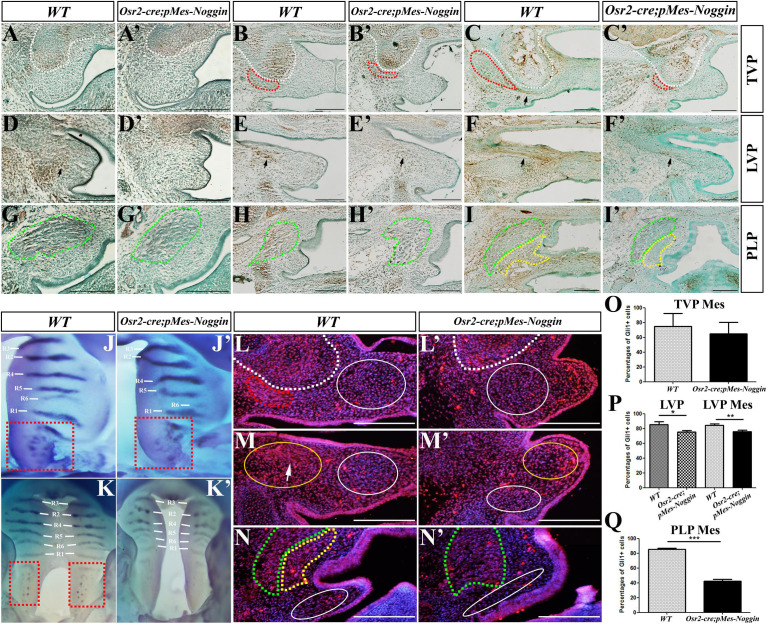
BMP-Smad and Shh-Gli1 signaling in the *Osr2-cre*^KI^*;pMes-Noggin* soft palates. **(A,A′–C,C′)** The immunostaining of p-Smad1/5/8 at the TVP levels of the E13.5 **(A)**, E14.5 **(B)**, and E16.5 **(C)** WT soft palates, and the E13.5 **(A′)**, E14.5 **(B′)**, and E16.5 **(C′)** *Osr2-cre*^KI^*;pMes-Noggin* soft palates. The arrow in **(C)** points to the p-Smad1/5/8 staining in TVP tendon. **(D,D′–F,F′)** The immunostaining of p-Smad1/5/8 at the LVP levels of the E13.5 **(D)**, E14.5 **(E)**, and E16.5 **(F)** WT soft palates, and the E13.5 **(D′)**, E14.5 **(E′**), and E16.5 **(F′**) *Osr2-cre*^KI^*;pMes-Noggin* soft palates. The arrows point to the LVP regions. **(G,G′–I,I′)** The immunostaining of p-Smad1/5/8 at the PLP levels of the E13.5 **(G)**, E14.5 **(H)**, and E16.5 **(I)** WT soft palates, and the E13.5 **(G′)**, E14.5 **(H′)**, and E16.5 **(I′)** *Osr2-cre*^KI^*;pMes-Noggin* soft palates. **(J,J′,K,K′)** Whole-mount *in situ* hybridization of *Shh* in the E13.5 **(J)** and E14.5 WT palates **(K)**, as well as the E13.5 **(J′)** and E14.5 *Osr2-cre*^KI^*;pMes-Noggin* palates **(K′)**. The R1–R6 delineate the *Shh-*expressing rugae, and the dashed red boxes circle soft palates with *Shh*-positive spots. **(L,L′–N,N′)** The Gli1 immunostaining in the E14.5 WT soft palate at the TVP **(L)**, LVP **(M)**, and PLP **(N)** levels, and the E14.5 *Osr2-cre*^KI^*;pMes-Noggin* soft palate at the TVP **(L′)**, LVP **(M′)**, and PLP **(N′)** levels. The white arrowheads in **(M)** delineate the LVP region. The white dotted lines delineate the boundary of HPP; the red dotted lines circle the TVP; the green dotted lines delineate the SPCs; the yellow dotted lines circle the PLPs; the white solid circles mean the mesenchymal areas for the statistical assay of the Gli1-positive percentages, while the yellow solid circles in **(M,M′)** mean the LVP areas for the counting of the Gli1-positive cells. Scale bars are 200 μm. **(O–Q)** The statistical assays of the percentages of the Gli1-positive cells in total cells at the E14.5 TVP levels **(O)**, LVP levels **(P),** and PLP levels **(Q)**. The percentage of Gli1-positive cells in the soft palate mesenchyme was 75.05% ± 17.05% and 65.03% ± 15.21% at the WT and *Osr2-cre*^KI^*;pMes-Noggin* TVP levels, respectively (*p* = 0.496; **O**). In the WT LVP myofibers, the percentage of Gli1-positive cells was 85.52% ± 3.83%; this percentage was reduced to 75.49% ± 1.77% in the *Osr2-cre*^KI^*;pMes-Noggin* TVP myofibers (*p* = 0.015; **P**). Similarly, in the mesenchyme at the LVP levels, the percentage of Gli1-positive cells in WT control was 84.50% ± 1.80%, which was significantly higher than the 75.69% ± 2.33% in the *Osr2-cre*^KI^*;pMes-Noggin* counterpart (*p* = 0.007; **P**). At PLP levels, the percentage of Gli1-positive cells was 85.22% ± 1.85% in WT mesenchyme, while it was 42.36% ± 2.31% in the *Osr2-cre*^KI^*;pMes-Noggin* mesenchyme (*p* = 0.0003; **Q**). *p < 0.05; **p < 0.01; ***p < 0.001. Three E14.5 *Osr2-cre*^KI^*;pMes-Noggin* embryos and their WT littermates were collected for statistical assay. BMP, bone morphogenetic protein; TVP, tensor veli palatini; LVP, levator veli palatini; PLP, palatopharyngeus; HPP, hypophosphatasia; SPC, superior pharyngeal constrictor.

The immunostaining of p-Smad1/5/8 was performed to verify the suppressed BMP-Smad signaling in the developing *Osr2-cre*^KI^*;pMes-Noggin* soft palate. At both the E13.5 WT and *Osr2-cre*^KI^*;pMes-Noggin* TVP levels, the p-Smad1/5/8 staining was only detected in the HPP, but excluded from the myogenic and mesenchymal compartments (Figures 2A,A′). At the E14.5 TVP levels, the p-Smad1/5/8 staining became robust in the HPP and still absent from both the WT and *Osr2-cre*^KI^*;pMes-Noggin* TVP and palatal mesenchyme (Figures 2B,B′). At the E15.5 TVP levels, though still robust in both the WT and *Osr2-cre*^KI^*;pMes-Noggin* HPPs, the p-Smad1/5/8 staining was also detected in the WT TVP tendon, but absent from the *Osr2-cre*^KI^*;pMes-Noggin* TVP tendon (Figures 2C,C′). In contrast, the p-Smad1/5/8 staining was noticeable in the E13.5, E14.5, and E15.5 WT LVP levels (Figures 2D–F), but significantly faded in the *Osr2-cre*^KI^*;pMes-Noggin* LVP areas (Figures 2D′–F′). At the E13.5 and E14.5 PLP levels, the p-Samd1/5/8 staining was mainly detected in the WT SPC myofibers (Figures 2G,H), while it was almost diminished in *Osr2-cre*^KI^*;pMes-Noggin* SPC (Figures 2G′,H′). At the E15.5 LVP levels, although the p-Smad1/5/8 staining was dramatically decreased in both the WT and *Osr2-cre*^KI^*;pMes-Noggin* SPCs and PLPs, the p-Smad1/5/8 staining in the WT mesenchyme was significantly stronger than in the *Osr2-cre*^KI^*;pMes-Noggin* mesenchyme (Figures 2I,I′).

*Shh* transcription was also suppressed in the epithelium of the *Osr2-cre*^KI^*;pMes-Noggin* hard and soft palates. In contrast to the six *Shh-*expressing rugae in the E13.5 WT hard palate (Figure 2J), although there were the six rugae detected, the *Shh* transcription in rugae 5 and 6 of the *Osr2-cre*^KI^*;pMes-Noggin* hard palate became significantly fainter (Figure 2J′). Consistently, the *Shh*-positive spots in the E13.5 *Osr2-cre*^KI^*;pMes-Noggin* soft palate became less and fainter than those in the WT counterpart (Figures 2J,J′). Such suppression became more evident it the E14.5 *Osr2-cre*^KI^*;pMes-Noggin* palates, in which not only the *Shh* expression in rugae obviously faded but also *Shh* spots in the soft palate were almost diminished (Figures 2K,K′). As a consequence of the reduced *Shh* transcription, the immunostaining of Gli1 in the E14.5 *Osr2-cre*^KI^*;pMes-Noggin* soft palate was remarkably decreased. At the TVP level, although the Gli1 staining was dramatically decreased in the *Osr2-cre*^KI^*;pMes-Noggin* TVP, the percentage of the Gli1-positive cells in the medial mesenchyme was comparable with that of the WT control (Figures 2L,L′,O). At the WT LVP level, the distribution of Gli1 staining was concentrated in the LVP and sparse in the medial mesenchyme (Figure 2M). In contrast, the distribution of Gli1 staining was much looser in both the *Osr2-cre*^KI^*;pMes-Noggin* LVP and the medial mesenchyme (Figure 2M′). Statistical assay indicated that the percentages of the Gli1-positive cells were significantly decreased in both the *Osr2-cre*^KI^*;pMes-Noggin* LVP and the mesenchyme (Figure 2P). Similarly, the Gli1 staining in the *Osr2-cre*^KI^*;pMes-Noggin* SPC, PLP, and mesenchyme, as well as the percentage of Gli1-positive cells in the *Osr2-cre*^KI^*;pMes-Noggin* PLP mesenchyme, was remarkably downregulated (Figures 2N,N′,Q). Taken together, the overexpressed *Noggin* inhibited the BMP-Smad signaling in the palatal muscles and mesenchyme of soft palate and disrupted *Shh* transcription in the epithelium of the *Osr2-cre*^KI^*;pMes-Noggin* soft palate.

### Decreased Cell Proliferation and Survival in *Osr2-cre*^KI^*;pMes-Noggin* Soft Palates

**FIGURE 3:**
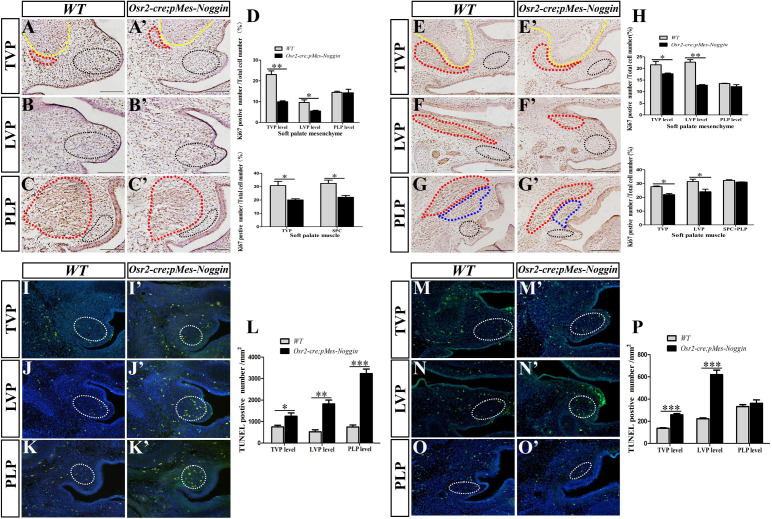
Ki67 immunostaining and TUNEL assay in *Osr2-cre*^KI^*;pMes-Noggin* soft palates. **(A,A′–C,C′)** The immunostaining of Ki67 in the E13.5 WT soft palate at the TVP **(A)**, LVP **(B)**, and PLP **(C)** levels, and the E13.5 *Osr2-cre*^KI^*;pMes-Noggin* soft palates at the TVP **(A′)**, LVP **(B′)**, and PLP **(C′)** levels. The yellow and red dotted lines in **(A,A′)** delineate the boundary of HPPs and TVPs, respectively. The red dotted lines in **(C,C′)** delineate the SPCs. The black dotted circles in **(A,A′–C,C′)** delineate the mesenchymal areas for Ki67-positive cell counting. **(D)** Statistical assay for the Ki67 percentages in the TVP and SPC myofibers, and mesenchyme in the E13.5 WT and *Osr2-cre*^KI^*;pMes-Noggin* soft palates. Student’s *t*-test showed that the Ki67 percentages in the E13.5 WT TVP (30.71% ± 5.59%) were higher than those in the *Osr2-cre*^KI^*;pMes-Noggin* TVP (20.04% ± 1.56%, *p* = 0.0335); the Ki67 percentages in the E13.5 WT SPC (32.46% ± 4.18%) were higher than those in the *Osr2-cre*^KI^*;pMes-Noggin* SPC (22.07% ± 2.39%, *p* = 0.0202); the Ki67 percentages in the E13.5 WT TVP mesenchyme (23.08% ± 3.09%) were higher than those in the *Osr2-cre*^KI^*;pMes-Noggin* TVP mesenchyme (9.95% ± 0.85%, *p* = 0.0019); the Ki67 percentages in the E13.5 WT LVP mesenchyme (9.615% ± 2.192%) were higher than those in the *Osr2-cre*^KI^*;pMes-Noggin* LVP mesenchyme (5.52% ± 0.604%, *p* = 0.0355); the Ki67 percentages in the E13.5 WT PLP mesenchyme (14.47% ± 0.936%) were comparable with those in the *Osr2-cre*^KI^*;pMes-Noggin* PLP mesenchyme (14.28% ± 3.104%, *p* = 0.92108) (four E13.5 *Osr2-cre*^KI^*;pMes-Noggin* embryos and their WT littermates were collected for statistical assay). **(E,E′–G,G′)** The immunostaining of Ki67 in the E14.5 WT soft palate at the TVP **(E)**, LVP **(F)**, and PLP **(G′)** levels, and the E14.5 *Osr2-cre*^KI^*;pMes-Noggin* soft palates at the TVP **(E′)**, LVP **(F′)**, and PLP **(G′)** levels. The yellow and red dotted lines in **(E**,**E′)** delineate the boundary of HPPs and TVPs, respectively. The red dotted lines in **(F,F′)** delineate the LVPs. The red dotted lines in **(G,G′)** delineate the SPCs. The blue dotted lines in **(G**,**G′)** circle PLPs. The black dotted circles in **(E,E′–G,G′)** delineate the mesenchymal areas for Ki67-positive cell counting. **(H)** Statistical assay for the Ki67 percentages in the TVP, LVP, SPC, and PLP myofibers and mesenchyme in the E14.5 WT and *Osr2-cre*^KI^*;pMes-Noggin* soft palates. Student’s *t*-test showed that the Ki67 percentages in the E14.5 WT TVP (27.90% ± 0.563%) were higher than those in the *Osr2-cre*^KI^*;pMes-Noggin* TVP (23.74% ± 3.765%, *p* = 0.0025); the Ki67 percentages in the E14.5 WT LVP (35.95% ± 9.36%) were higher than those in the *Osr2-cre*^KI^*;pMes-Noggin* SPC (25.69% ± 4.21%, *p* = 0.04357); the Ki67 percentages in the E14.5 WT SPC and PLP (31.67% ± 1.362%) were indistinguishable from those in the *Osr2-cre*^KI^*;pMes-Noggin* SPC and PLP (30.71% ± 0.78%, *p* = 0.2561); the Ki67 percentages in the E14.5 WT TVP mesenchyme (23.55% ± 4.16%) were higher than those in the *Osr2-cre*^KI^*;pMes-Noggin* TVP mesenchyme (18.49% ± 1.466%, *p* = 0.0121); the Ki67 percentages in the E14.5 WT LVP mesenchyme (21.03% ± 3.725%) were higher than those in the *Osr2-cre*^KI^*;pMes-Noggin* LVP mesenchyme (13.19% ± 0.844%, *p* = 0.00638); the Ki67 percentages in E14.5 WT PLP mesenchyme (14.52% ± 2.06%) were similar to those in the *Osr2-cre*^KI^*;pMes-Noggin* PLP mesenchyme (12.72% ± 1.654%, *p* = 0.22456). Five E14.5 *Osr2-cre*^KI^*;pMes-Noggin* embryos and their WT littermates were collected for statistical assay. **(I,I′–K,K′)** The TUNEL assay in the E13.5 WT soft palate at the TVP **(I)**, LVP **(J)**, and PLP **(K)** levels, and the E13.5 *Osr2-cre*^KI^*;pMes-Noggin* soft palates at the TVP **(I′)**, LVP **(J′)**, and PLP **(K′)** levels. The white dotted circles in **(I,I′–K,K′)** mean the areas for TUNEL-positive cell counting. **(L)** Statistical assay for the TUNEL densities in the E13.5 WT and *Osr2-cre*^KI^*;pMes-Noggin* soft palates at the TVP, LVP, and PLP levels. Student’s *t*-test showed that the TUNEL densities in the E13.5 WT TVP level (754.29 ± 139.14 cells/mm^2^) were much lower than those in the *Osr2-cre*^KI^*;pMes-Noggin* TVP level (1,251.18 ± 269.98 cells/mm^2^, *p* = 0.0472); the TUNEL densities in the E13.5 WT LVP level (526.23 ± 155.39 cells/mm^2^) were lower than those in the *Osr2-cre*^KI^*;pMes-Noggin* LVP level (1,823.57 ± 309.96 cells/mm^2^, *p* = 0.00292); the TUNEL densities in the E13.5 WT PLP level (746.05 ± 8.01 cells/mm^2^) were lower than those in the *Osr2-cre*^KI^*;pMes-Noggin* PLP level (3,332.16 ± 366.88 cells/mm^2^, *p* = 0.000435). Three E13.5 *Osr2-cre*^KI^*;pMes-Noggin* embryos and their WT littermates were collected for statistical assay. **(M,M′–O,O′)** The TUNEL assay in the E14.5 WT soft palate at the TVP **(M)**, LVP **(N)**, and PLP **(O)** levels, and the E13.5 *Osr2-cre*^KI^*;pMes-Noggin* soft palates at the TVP **(M′)**, LVP, **(N′)** and PLP **(O′)** levels. The white dotted circles in **(M,M′–O,O′)** mean the areas for TUNEL-positive cell counting. **(P)** Statistical assay for the TUNEL densities in the E14.5 WT and *Osr2-cre*^KI^*;pMes-Noggin* soft palates at the TVP, LVP, and PLP levels. Student’s *t*-test showed that the TUNEL densities in the E14.5 WT TVP level (136.65 ± 8.41 cells/mm^2^) were much lower than those in the *Osr2-cre*^KI^*;pMes-Noggin* TVP level (259.01 ± 17.89 cells/mm^2^, *p* = 0.0004275); the TUNEL densities in the E14.5 WT LVP level (222.01 ± 14.21 cells/mm^2^) were significantly lower than those in the *Osr2-cre*^KI^*;pMes-Noggin* LVP level (620.29 ± 70.66 cells/mm^2^, *p* = 0.000666); the TUNEL densities in the E14.5 WT PLP level (323.08 ± 13.34 cells/mm^2^) were comparable with those in the *Osr2-cre*^KI^*;pMes-Noggin* PLP level (362.25 ± 51.88 cells/mm^2^, *p* = 0.2740). ^∗^*p* < 0.05; ^∗∗^*p* < 0.01; ^∗∗∗^*p* < 0.001. Four E14.5 *Osr2-cre*^KI^*;pMes-Noggin* embryos and their WT littermates were collected for statistical assay. TVP, tensor veli palatini; LVP, levator veli palatini; PLP, palatopharyngeus; HPP, hypophosphatasia; SPC, superior pharyngeal constrictor.

To explore how the *Osr2-cre*^KI^*;pMes-Noggin* soft palatal shelves were reduced in size, the cell proliferation and apoptosis were examined by Ki76 immunostaining and TUNEL assay. The percentages of the Ki67-positive nucleus in the E13.5 *Osr2-cre*^KI^*;pMes-Noggin* TVP and SPC were significantly lower than those in the WT controls (Figures 3A,A′,C,C′,D). Although comparable with those at the WT PLP level (Figures 3C,C′,D), the percentages of the Ki67-positive nucleus in the mesenchyme of E13.5 *Osr2-cre*^KI^*;pMes-Noggin* soft palate were significantly decreased at the TVP and LVP levels (Figures 3A,A′,B,B′,D). Such tendency became more evident at E14.5, in that the percentages of Ki67-positive nucleus were decreased in both the muscles and mesenchyme at the *Osr2-cre*^KI^*;pMes-Noggin* TVP and LVP levels (Figures 3E,E′,F,F′,H), while both the muscles and mesenchyme at the E14.5 *Osr2-cre*^KI^*;pMes-Noggin* PLP levels displayed comparable percentages with the WT controls (Figures 3G,G′,H).

On the other hand, TUNEL assay indicated that the densities of the cell apoptosis at the TVP, LVP, and PLP levels in the E13.5 *Osr2-cre*^KI^*;pMes-Noggin* soft palate were all statistically higher than those in the E13.5 WT soft palates (Figures 3I–K′,L). Even in the E14.5, the *Osr2-cre*^KI^*;pMes-Noggin* soft palate exhibited significantly higher densities of cell apoptosis at the TVP and LVP levels (Figures 3M,M′,N,N′,P), though comparable with the WT counterparts at the PLP level (Figures 3O,O′,P). These findings suggested that there were reduced cell proliferation and survival in the *Osr2-cre*^KI^*;pMes-Noggin* soft palatal shelves, which could primarily lead to the cleft in soft palate.

### Impaired Aponeurosis and Tendon Development in the *Osr2-cre*^KI^*;pMes-Noggin* Soft Palate

**FIGURE 4:**
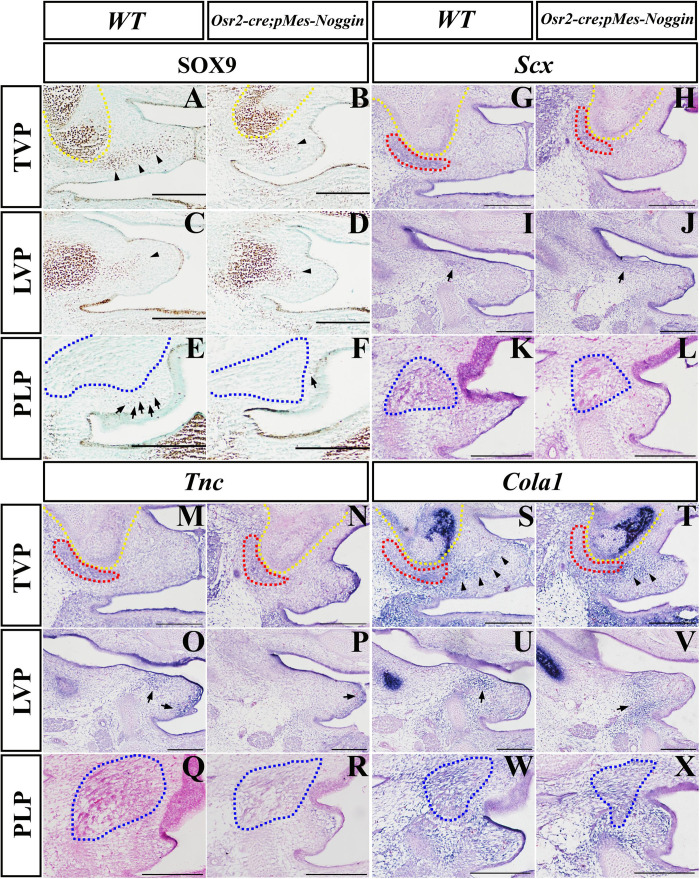
The Sox9, *Scx*, *TnC*, and *Col1a1* expression in *Osr2-cre*^KI^*;pMes-Noggin* soft palates. **(A–F)** The immunostaining of Sox9 in the E14.5 WT soft palate at the TVP **(A)**, LVP **(C)**, and PLP **(E)** levels, and the E14.5 *Osr2-cre*^KI^*;pMes-Noggin* soft palate at the TVP **(B)**, LVP **(D)**, and PLP **(F)** levels. The arrowheads in **(A–D)** point to the Sox9 domain in the mesenchyme. The arrows in **(E,F)** point to the Sox9-positive cells in the mesenchyme. **(G–L)** *In situ* hybridization of *Scx* in the E14.5 WT soft palate at the TVP **(G)**, LVP **(I)**, and PLP **(K)** levels, and the E14.5 *Osr2-cre*^KI^*;pMes-Noggin* soft palate at the TVP **(H)**, LVP **(J)**, and PLP **(L)** levels. The arrows in **(I,J)** point to the LVP areas. **(M–R)** *In situ* hybridization of *TnC* in the E14.5 WT soft palate at the TVP **(M)**, LVP **(O)**, and PLP **(Q)** levels, and the E14.5 *Osr2-cre*^KI^*;pMes-Noggin* soft palate at the TVP **(N)**, LVP **(P)**, and PLP **(R**) levels. The arrows in **(O,P)** point to the *TnC*-positive areas. **(S–X)** *In situ* hybridization of *Col1a1* in the E14.5 WT soft palate at the TVP **(S)**, LVP **(U)**, and PLP **(W)** levels, and the E14.5 *Osr2-cre*^KI^*;pMes-Noggin* soft palate at the TVP **(T)**, LVP **(V)**, and PLP **(X)** levels. The arrowheads in **(S,T)** delineate the *Col1a1*-positive areas along the presumptive aponeurosis. The arrows in **(U,V)** point to the *Col1a1*-positive areas. The yellow dotted lines delineate the HPPs; the red dotted lines circle TVPs; the blue dotted lines delineate SPCs and PLPs. Scale bars are 200 μm. TVP, tensor veli palatini; LVP, levator veli palatini; PLP, palatopharyngeus; HPP, hypophosphatasia; SPC, superior pharyngeal constrictor.

During craniofacial development, Sox9 acts as the marker of the osteo-chondrogenic mesenchyme (Almalki and Agrawal, 2016; Dash and Trainor, 2020). At the E14.5 WT TVP level, the robust Sox9 expression was detected not only in the HPP but also in the mesenchyme of the presumptive aponeurosis, which connected the bilateral palatal shelves (Figure 4A). In contrast, the Sox9-expressing domain in the *Osr2-cre*^KI^*;pMes-Noggin* soft palate was dramatically reduced in the mesenchyme for the presumptive aponeurosis, though differed little from the WT control in the HPP (Figure 4B). Similar to the TVP level, the Sox9 staining at the LVP showed no discrepancy between the WT and *Osr2-cre*^KI^*;pMes-Noggin* posterior HPP (Figures 4C,D), but in the presumptive *Osr2-cre*^KI^*;pMes-Noggin* aponeurosis, the Sox9-expressing area was also greatly decreased than in the WT control (Figures 4C,D). At the PLP level, the Sox9 expression was only sporadically distributed in the WT mesenchyme (Figures 4E,F), which was also evidently decreased in the *Osr2-cre*^KI^*;pMes-Noggin* mesenchyme (Figures 4E,F). These data implicated an attenuated osteo-chondrogenic fate of the aponeurosis in the *Osr2-cre*^KI^*;pMes-Noggin* soft palate. ISH showed that the transcription of *Scx*, the key transcription factor for tenogenic specification and maintenance, was suppressed moderately in the E14.5 *Osr2-cre*^KI^*;pMes-Noggin* TVP and mildly in the LVP compared with the WT counterparts (Figures 4G–J). There was no *Scx* transcription detected in neither the WT nor *Osr2-cre*^KI^*;pMes-Noggin* PLP levels (Figures 4K,L). At the E14.5 TVP and LVP levels, the extracellular matrix, *Tnc* was also transcribed more weakly in both the *Osr2-cre*^KI^*;pMes-Noggin* myogenic and mesenchymal areas than those in the WT controls (Figures 4M–P). Similar to the *Scx* expression, there was no *Tnc* expression detected in the WT and *Osr2-cre*^KI^*;pMes-Noggin* PLP levels (Figures 4Q,R). Compared with the WT soft palate (Figures 4S,U,W), the *Col1α1-*expressing domains in the myogenic and aponeurosis areas of the E14.5 *Osr2-cre*^KI^*;pMes-Noggin* soft palate were remarkably decreased at the TVP and LVP levels (Figures 4T,V), but almost indistinguishable at the PLP level (Figure 4X). These results suggested that both the specification and differentiation of the tendons and aponeurosis in *Osr2-cre*^KI^*;pMes-Noggin* soft palate were impacted by the overexpressed *Noggin.*

### Overexpressed *Noggin* in Myoblasts Mildly Affected the Fusion of Soft Palate

**FIGURE 5:**
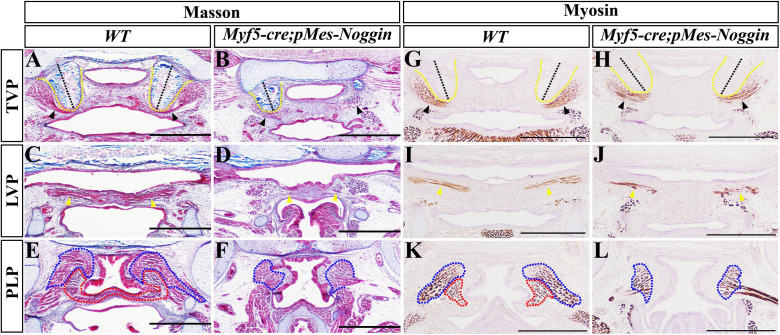
The fusion and muscle morphology in *Myf5-cre;pMes-Noggin* soft palate. **(A–F)** Masson staining of the E17.5 WT and *Myf5-cre;pMes-Noggin* soft palates. The TVP **(A)**, LVP **(C)**, and PLP **(E)** levels of WT soft palate were compared with the *Myf5-cre;pMes-Noggin* TVP **(B)**, LVP **(D),** and PLP **(F)** levels. **(G–L)** The immunostaining of myosin of the E16.5 WT and *Myf5-cre;pMes-Noggin* soft palates. The TVP **(G)**, LVP **(I)**, and PLP **(K)** of WT soft palate were compared with the TVP **(H)**, LVP **(J)**, and PLP **(L)** of the *Myf5-cre;pMes-Noggin* soft palate. The black dashed lines indicate the orientation of HPPs; the black arrowheads delineate TVPs; the yellow arrowheads point to LVPs; the blue dotted lines circle SPCs; the red dotted lines mark PLPs. Scale bars are 200 μm. TVP, tensor veli palatini; LVP, levator veli palatini; PLP, palatopharyngeus; HPP, hypophosphatasia; SPC, superior pharyngeal constrictor.

To further explore the impacts of Noggin on palatal myogenesis and palatogenesis, the *Noggin* transgene was activated in myoblasts by *Myf5-cre*. Histological sections indicated that compared with the WT controls (Figures 5A,C,E), the E17.5 *Myf5-cre;pMes-Noggin* soft palatal shelves had fused into an integral plate at the TVP and LVP levels (Figures 5B,D), but still separated from each other at the PLP level (Figure 5F). Intriguingly, the *Myf5-cre;pMes-Noggin* HPP was orientated introversive as the WT control did (Figures 5A,B), instead of extraversive in the *Osr2-cre*^KI^*;pMes-Noggin* soft palate (Figures 1B,D). The myosin immunostaining in the E16.5 soft palates disclosed that the *Myf5-cre;pMes-Noggin* TVP, LVP, and SPC myofibers (Figures 5H,J) were reduced and sparser greatly than those in the WT controls (Figures 5G,I), and the *Myf5-cre;pMes-Noggin* PLP was even diminished (Figures 5K,L), suggesting a more severe hypoplasia than the *Osr2-cre*^KI^*;pMes-Noggin* PLP (Figure 1P). Taken together, these results indicated that the dramatically compromised myogenesis in the *Myf5-cre;pMes-Noggin* soft palate had no effect on the fusion at the TVP and LVP levels, but disrupted the fusion at the PLP level.

### Suppressed Tenogenic Gene Expression in *Myf5-cre;pMes-Noggin* Soft Palate

**FIGURE 6:**
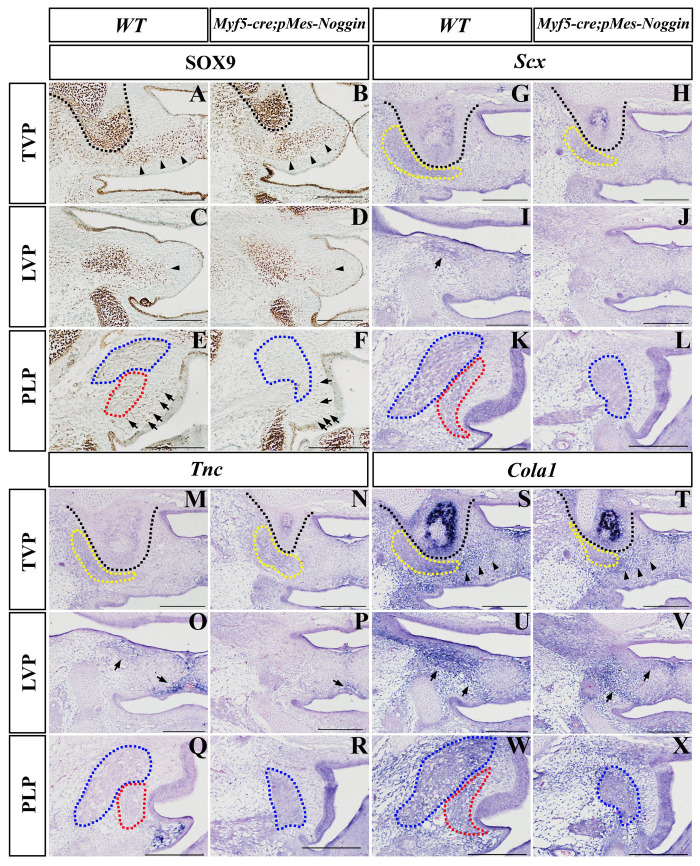
The Sox9, *Scx*, *TnC*, and *Col1a1* expression in *Myf5-cre;pMes-Noggin* soft palates. **(A–F)** The Sox9 immunostaining in the E14.5 WT soft palate at the TVP **(A)**, LVP **(C)**, and PLP **(E)** levels, and the E14.5 *Myf5-cre;pMes-Noggin* soft palate at the TVP **(B)**, LVP **(D)**, and PLP **(F)** levels. The arrowheads in **(A–D)** point to the Sox9 domain in the mesenchyme. The arrows in **(E,F)** point to the Sox9-positive cells in the mesenchyme. **(G–L)** *In situ* hybridization of *Scx* in the E14.5 WT soft palate at the TVP **(G)**, LVP **(I)**, and PLP **(K)** levels, and the E14.5 *Osr2-cre*^KI^*;pMes-Noggin* soft palate at the TVP **(H)**, LVP **(J)**, and PLP **(L)** levels. The arrows in **(I)** point to the LVP areas. **(M–R)** *In situ* hybridization of *TnC* in the E14.5 WT soft palate at the TVP **(M)**, LVP **(O)**, and PLP **(Q)** levels, and the E14.5 *Osr2-cre*^KI^*;pMes-Noggin* soft palate at the TVP **(N)**, LVP **(P)**, and PLP **(R)** levels. The arrows in **(O,P)** point to the *TnC*-positive area. **(S–X)** *In situ* hybridization of *Col1a1* in the E14.5 WT soft palate at the TVP **(S)**, LVP **(U)**, and PLP **(W)** levels, and the E14.5 *Osr2-cre*^KI^*;pMes-Noggin* soft palate at the TVP **(T)**, LVP **(V)**, and PLP **(X)** levels. The arrowheads in **(S,T)** delineate the *Col1a1*-positive areas along the presumptive aponeurosis. The arrows in **(U,V)** point to the *Col1a1*-positive areas. The black dotted lines delineate the HPPs; the yellow dotted lines circle TVPs; the blue dotted lines delineate SPCs; the red dotted lines delineate PLPs. Scale bars are 200 μm. TVP, tensor veli palatini; LVP, levator veli palatini; PLP, palatopharyngeus; HPP, hypophosphatasia; SPC, superior pharyngeal constrictor.

Immunohistochemistry displayed that at the TVP levels, the Sox9 expression in both the HPP and the aponeurosis of the E14.5 *Myf5-cre;pMes-Noggin* soft palate was comparable with that in the WT control (Figures 6A,B). At the LVP level, the Sox9-expressing area in the *Myf5-cre;pMes-Noggin* aponeurosis was mildly reduced than in the WT control (Figures 6C,D), while in the PLP level, the number of the Sox9-positive mesenchymal cells was obviously less than that in the WT PLP mesenchyme (Figures 6E,F). ISH was performed to check the expression of *Scx*, *Tnc*, and *Col1a1*. At the E14.5 TVP level, the *Scx* expression in the *Myf5-cre;pMes-Noggin* TVP region was comparable with that in the corresponding area in the WT control (Figures 6G,H). However, the *Scx* transcription in the E14.5 *Myf5-cre;pMes-Noggin* LVP level was almost diminished (Figures 6I,J). And there was no *Scx* transcription detected in the E14.5 WT and *Myf5-cre;pMes-Noggin* PLP levels (Figures 6K,L). Similar to *Scx* expression, the *Tnc* transcription in the E14.5 *Myf5-cre;pMes-Noggin* TVP and LVP areas was also diminished and also decreased significantly in the medial mesenchyme (Figures 6M–P). The *Tnc* expression was detected in the mesenchyme of the E14.5 WT PLP level, which disappeared in the *Myf5-cre;pMes-Noggin* PLP level (Figures 6Q,R). Additionally, although the *Col1a1*-expressing domains at the E14.5 *Myf5-cre;pMes-Noggin* TVP and LVP areas were reduced (Figures 6S,U), the domains along the aponeurosis were similar to those in the WT controls (Figures 6T,V). The *Col1a1* transcription in the mesenchyme at the WT PLP level was obviously more robust than that in the mesenchyme of the *Myf5-cre;pMes-Noggin* PLP level (Figures 6W,X). These results suggested that in the *Myf5-cre;pMes-Noggin* soft palate, the tenogenesis was impaired severely as that in the *Osr2-cre;pMes-Noggin* soft palate, but the aponeurosis development was affected moderately.

### Abrogation of Myoblasts Affected Little the Fusion of Soft Palate

**FIGURE 7:**
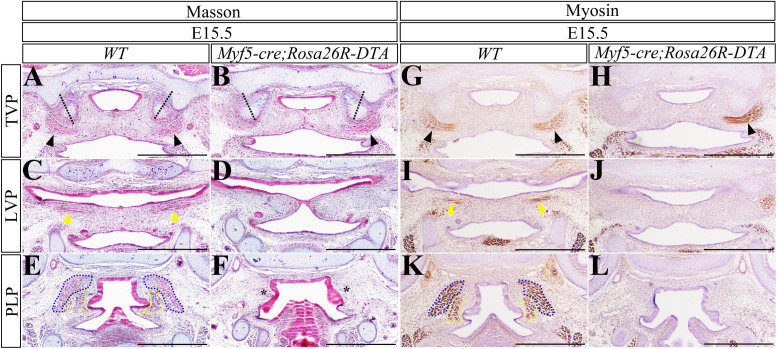
The fusion of *Myf5-cre; Rosa26R-DTA* soft palate. **(A–F)** Masson staining of the E15.5 WT and *Myf5-cre; Rosa26R-DTA* soft palates. The WT TVP **(A)**, LVP **(C)**, and PLP **(E)** levels were compared with the *Myf5-cre;pMes-Noggin* TVP **(B)**, LVP **(D)**, and PLP **(F)** levels. **(G–L)** The immunostaining of myosin of the E15.5 WT and *Myf5-cre; Rosa26R-DTA* soft palates. The WT TVP **(G)**, LVP **(I)**, and PLP **(K)** were compared with the *Myf5-cre;pMes-Noggin* TVP **(H)**, LVP **(J)**, and PLP **(L)** levels. The black dashed lines indicate the orientation of HPPs; the black arrowheads delineate to TVPs; the yellow arrowheads point to LVPs; the blue dotted lines circle SPCs; the yellow dotted lines mark PLPs. Scale bars are 200 μm. TVP, tensor veli palatini; LVP, levator veli palatini; PLP, palatopharyngeus; HPP, hypophosphatasia; SPC, superior pharyngeal constrictor.

To confirm that the myogenesis is dispensable for the palatal fusion, a conditional transgene encoding diphtheria toxin A subunit (DTA) in myoblasts was activated by *Myf5-cre* to abrogate the palatal muscles. The *Myf5-cre;Rosa26R-DTA* mouse embryos died approximately at E15.5, when the WT soft palates had been completely integrated at both the TVP and LVP levels, but were still separated at the PLP level (Figures 7A,C). In contrast, almost all the *Myf5-cre; Rosa26R-DTA* soft palates were fusing with the degenerating epithelial beam at the TVP level (13 cases in 14 mutants; Figure 7B). Interestingly, the fused *Myf5-cre;Rosa26R-DTA* soft palate exhibited an intraversive HPP, similar to the WT control (Figures 7A,B). At the LVP level, 50% of the *Myf5-cre; Rosa26R-DTA* soft palates were fusing, and the other 50% just had the epithelial contact (Figure 7D). At the PLP level, both the WT and *Myf5-cre;Rosa26R-DTA* mice displayed separated soft palates, while the *Myf5-cre;Rosa26R-DTA* palatal shelves were much smaller than those of the WT controls (Figures 7E,F). Myosin immunostaining confirmed that compared with the E15.5 WT soft palate (Figures 7G,I,K), the *Myf5-cre;Rosa26R-DTA* soft palates were devoid of TVP, LVP, PLP, and SPC (Figures 7H,J,L), though the unilateral TVP was preserved in some instances (Figure 7H). This finding confirmed that the TVP and LVP were dispensable for the fusion of soft palate.

## Discussion

### The Overexpressed *Noggin* in Palatal Mesenchyme Impaired Myogenesis and Tenogenesis by Suppressing BMP-Smad Signaling

During the soft palatogenesis in mouse, *Bmp3* was activated in the medial mesenchyme of the middle and posterior palates (Fu et al., 2017). Our study suggested that BMP-Smad signaling was activated in the HPP and the mesenchyme surrounding TVP, LVP, SPC, and PLP. The overexpressed *Noggin* by *Osr2-cre*^KI^ suppressed the BMP-Smad signaling severely in the mesenchyme surrounding palatal muscles, but mildly in the HPP. These results suggested that similar to Wnt/β-catenin, FGF, and Hh signaling, the BMP-Smad signaling in the palatal mesenchyme also played a role in the myogenesis of soft palates (Janečková et al., 2019). Previous studies demonstrated that although initiated independently, the developing muscles and tendons require reciprocal interactions for their differentiation and maturation (Grenier et al., 2009). The decreased *Scx*, *Tnc*, and *Col1a1* transcription in the *Osr2-cre*^KI^*;pMes-Noggin* palatal muscle regions suggested that the differentiation of the mesenchyme-derived tendons and connective tissues was impaired by the overexpressed *Noggin*. Since BMP-Smad signaling was activated at the interface between muscles and tendons, and BMPs promoted the specification of tendon stem cells and tendon–bone integration (Wang et al., 2010; Schwarting et al., 2016; Orfei et al., 2019), the BMP-Smad signaling suppressed by *Noggin* overexpression could interrupt the tendon differentiation and maturation in the *Osr2-cre*^KI^*;pMes-Noggin* soft palate, which was consistent with the decreased *Scx*, *Tnc*, and *Col1* transcription. Theoretically, the Noggin secreted by mesenchymal cells can inhibit BMP signaling in both the mesenchymal cells and myoblasts of soft palate. A previous study demonstrated that although suppression on BMP signaling was essential for the myogenic induction of both the somite- and cranial mesoderm-derived muscles at early embryonic stages (Tzahor et al., 2003; Borok et al., 2020), BMP signaling maintained the satellite cells in the proliferating status at the late embryonic and postnatal stages, and thus, suppression on BMP signaling led to muscle atrophy (Wang et al., 2010; Friedrichs et al., 2011; Stantzou et al., 2017). These reports coincided with our findings that the muscle hypoplasia in the *Myf5-cre;pMes-Noggin* soft palate was much more severe than that in the *Osr2-cre*^KI^*;pMes-Noggin* soft palate. Therefore, the muscle hypoplasia in the *Osr2-cre*^KI^*;pMes-Noggin* soft palate was preferentially attributed to the impaired tenogenic differentiation and maturation. Intriguingly, Noggin was reported to induce *Scx* transcription during the early specification of limb tendons (Schweitzer et al., 2001), while in this study, the overexpressed *Noggin* inhibited the tenogenesis. The contradictory phenomena may result from the signaling other than BMP-Smad signaling, which was altered by the overexpressed *Noggin* (Ohki et al., 2020). Actually, the decreased *Scx* transcription in both the *Osr2-cre*^KI^*;pMes-Noggin* and *Myf5-cre;pMes-Noggin* soft palates was supposed to be regarded as a sign of the impaired tenogenic differentiation and maturation, because *Scx* was also essential for the maintenance of tendons.

### The Overexpressed *Noggin* Impaired Cell Proliferation and Survival by Suppressing Epithelial *Shh* Expression

In the developing hard palate, the BMPs secreted from mesenchyme activates *Shh* expression in the epithelium, which maintains cell proliferation and survival by activating the expression of *Bmp4* and *Fgf10* in the underlying palatal mesenchyme through Smoothen-Gli1 pathway (Zhang et al., 2002; Lan and Jiang, 2009; Zhou et al., 2013). Thus, we explored whether the Shh signaling was also involved in the cell proliferation and survival during soft palatogenesis. In the *Osr2-cre*^KI^*;pMes-Noggin* mouse, the diminished *Shh* expression in the epithelium of both the hard and soft palates, as well as the attenuated Gli1 activity in the soft palatal mesenchyme, indicated an interrupted interaction between the soft palatal epithelium and mesenchyme. Combined with the decreased cell proliferation and increased cell death in the mesenchyme of the *Osr2-cre*^KI^*;pMes-Noggin* soft palate, it also suggested that the Bmp-Shh-Gli1 regulatory network was essential for the cell proliferation and survival of soft palatal mesenchyme, which is similar to that in the developing hard palate. Moreover, since the sufficient amount of cells is the prerequisite for the connection and fusion of palatal shelves in both the hard and soft palates (Dixon et al., 2011; Bush and Jiang, 2012), the cleft of the *Osr2-cre*^KI^*;pMes-Noggin* soft palates resulted primarily from the reduced palatal shelves at least partially.

### Myogenesis and Tenogenesis Are Dispensable for the Fusion of Soft Palate

The patterning, morphogenesis, and differentiation of palatal muscles were essential for the normal function of soft palates (Li et al., 2017). It is believed that the mesenchymal clues instruct the migration, patterning, and differentiation of myoblasts in the developing soft palates (Iwata et al., 2014; Janečková et al., 2019). However, whether the mesenchymal or myogenic defects in soft palate lead to cleft soft palate is still under debate. Compared with the reduced palatal shelves and hypoplastic muscles in the *Osr2-cre*^KI^*;pMes-Noggin* soft palate, the *Myf5-cre;pMes-Noggin* palatal muscles exhibited a much more severe hypoplasia, especially in the SPC and PLP, even though the *Myf5-cre;pMes-Noggin* palatal shelves fused at the TVP and LVP levels, and only a fissure was detected in the PLP level. This finding was confirmed by the fused soft palate in the E15.5 *Myf5-cre;Rosa26R-DTA* mice in which the TVP, LVP, SPC, and PLP were all eliminated. Thus, it is suggested that the myogenesis is dispensable for the fusion of soft palate at the TVP and LVP levels, but required at the PLP level.

The *Myf5-cre;pMes-Noggin* palatal muscles were evidently compromised directly by the Noggin secreted from myoblasts. Similar to the *Osr2-cre*^KI^*;pMes-Noggin* soft palate, the noticeable downregulated transcription of *Scx*, *Tnc*, and *Col1a1* was also detected in the *Myf5-cre;pMes-Noggin* TVP, LVP, and SPC regions, verifying the requirements on the interactions between the developing craniofacial tendons and muscles (Grenier et al., 2009). In contrast, the Sox9, *Tnc*, and *Col1a1* expression in the medial mesenchyme of *Myf5-cre;pMes-Noggin* soft palate was only slightly affected, suggesting that the medial mesenchyme of soft palate, which developed into the aponeurosis, was critical for the soft palate fusion. These findings also implicated that even in an intact palate, the dysfunction of soft palate could also take place because of the compromised myogenesis or tenogenesis.

### The Mis-Oriented Hypophosphatasia Resulted From the Cleft in Soft Palate

Since the palatal fusion is initiated in the anterior secondary palate and, then, extend forwardly to the primary palate and backwardly to the soft palate (Bush and Jiang, 2012), it raised a speculation that the cleft in soft palate might result secondarily from the cleft in the hard palate. This notion is enforced by the few cases of the cleft hard palate (cleft in the hard palate with the intact soft palate) in both humans and mice (Dixon et al., 2011). Our and other researchers’ studies demonstrated that the interruption on TGFβ/BMP signaling through *Wnt1-cre* or *Osr2-cre* noticeably impaired the morphogenesis and differentiation of palatal bones (Baek et al., 2011; Parada et al., 2013; Li et al., 2021), which implied that the deformed palatine (especially the HPP) could cause cleft soft palate by disrupting the pattern of palatal muscles. The extroversive HPP in *Osr2-cre*^KI^*;pMes-Noggin* soft palate also implicated the correlation between HPP deformity and cleft soft palate. However, the *Osr2-cre*^KI^*;pMes-Noggin* palatal muscles showed no patterning defect, and the HPPs in *Myf5-cre;pMes-Noggin* and *Myf5-cre;Rosa26R-DTA* soft palates were intraversive, suggesting that the orientation of HPP was determined not by the muscles but by the fusion of soft palate. We speculated that in the fused soft palate, the HPP would be pulled intraversive by the contraction of the contralateral palatal shelves. Thus, the mis-oriented HPP in *Osr2-cre*^KI^*;pMes-Noggin* soft palate was not the cause but the consequence of cleft soft palate.

### The Aponeurosis Development Is Critical for the Fusion of Soft Palates

The integrity of the hard palate is achieved by the fused maxilla and palatine, while the integrity of soft palate is accomplished by forming an intact aponeurosis. In this study, Sox9 expression was found in the presumptive aponeurosis, but reduced in the *Osr2-cre*^KI^*;pMes-Noggin* soft palate. Because BMP signaling balanced the *Noggin* and *Sox9* expression during skeletogenesis (Nifuji and Noda, 1999; Zehentner et al., 1999), both the overexpressed *Noggin* and suppressed BMP-Smad signaling could downregulate Sox9 expression, through which attenuated the osteo-chondrogenic specification of the aponeurosis. Similarly, the decreased *Tnc* and *Col1a1* transcription in the medial mesenchyme also suggested the suppressed differentiation of the *Osr2-cre*^KI^*;pMes-Noggin* aponeurosis. Additionally, the little impacted Sox9, *TnC*, and *Col1* expression in the medial mesenchyme of *Myf5-cre;pMes-Noggin* soft palate supported the notion that the aponeurosis specification and differentiation were critical for the fusion of soft palate. Actually, even in the plastic surgery for cleft soft palates or submucous cleft palates, the extension of aponeurosis by Zig-Zag incision and suture, instead of the bilateral LVP connection, was critical for the re-building of soft palate (Losee and Kirschner, 2009), which suggested the significance of the sufficient volume of the aponeurosis in the fusion and integrity of soft palate.

In summary, we investigated the defects in the soft palate of *Osr2-cre*^KI^*;pMes-Noggin* mice to address the impacts of the suppressed BMP signaling on the development of soft palate. The *Noggin* overexpression in palatal mesenchyme mainly inhibited the differentiation of aponeurosis and tendons, which resulted in the hypoplasia of palatal muscles. The overexpressed *Noggin* also decreased the mesenchymal cell proliferation and survival by disrupting epithelial *Shh* expression, which reduced the size of soft palatal shelves. Although the myogenesis and tenogenesis were critical for the function of soft palate, they were dispensable for the fusion of soft palate. On the contrary, the cleft in the *Osr2-cre*^KI^*;pMes-Noggin* soft palate was attributed to the reduced cell proliferation and survival caused by the interrupted Shh-Gli1 signaling, and the impaired specification and differentiation of aponeurosis.

## Data Availability Statement

The original contributions presented in the study are included in the article/Supplementary Material, further inquiries can be directed to the corresponding author/s.

## Ethics Statement

The animal study was reviewed and approved by The Laboratory Animal Ethics Committee at Dalian Medical University.

## Author Contributions

CL and JX conceived the studies and wrote the manuscript. XC, BW, HL, LZ, and WC designed the methodology and supervised the study. SW, JD, and NL conducted the experiments and acquired and analyzed the data. All the authors contributed to the article and approved the submitted version.

## Conflict of Interest

The authors declare that the research was conducted in the absence of any commercial or financial relationships that could be construed as a potential conflict of interest.

## Publisher’s Note

All claims expressed in this article are solely those of the authors and do not necessarily represent those of their affiliated organizations, or those of the publisher, the editors and the reviewers. Any product that may be evaluated in this article, or claim that may be made by its manufacturer, is not guaranteed or endorsed by the publisher.

## Supplementary Material

Supplementary Figure 1 The fluorescence pattern in *Osr*2-cre^Ki^; Rosa26R-mT/mG soft palate. **(A)** The Tvp level of soft palate in E16.5 *Osr*2-cre^Ki^; Rosa26R-mT/mG mouse. White dotted line delineated aponeurosis; asterisk meant Hpp; the white arrows pointed to the Tvp tendons; white arrowheads pointed to Tvps. **(B)** The Lvp level in E16.5 *Osr*2-cre^Ki^; Rosa26R-mT/mG soft palate. White arrowheads delineated Lvp myofibers; asterisk meant the aponeurosis. **(C)** The Plp level in E16.5 *Osr*2-cre^Ki^; Rosa26R-mT/mG soft palate. White arrowheads delineated Plp myofibers; yellow arrowheads pointed to Spc myofibers; asterisk meant the connective tissue. (Tvp, tensor veli palatini; Lvp, levator veli palatini; Plp, palatopharyngeus).

Supplementary Figure 2 The negative controls of Tunel assay. The Lvp levels in E14.5 Wt **(A)** and *Osr*2-cre^Ki^; pMes-Noggin soft palate **(B)**. In both **(A,B)**, the Tunel procedure followed the instruction in the *In Situ* Cell Death Detection Kit, except the solution of Blue Cap 1 was not added into the samples, but replaced by distilled water. So the TdT enzyme was left out, and no signal (green fluorescence) was detected.
