# Supplementary figures and images for: Noggin Overexpression Impairs the Development of Muscles, Tendons, and Aponeurosis in Soft Palates by Disrupting BMP-Smad and Shh-Gli1 Signaling
Source: Front Cell Dev Biol. 2021 Sep 7;9:711334. doi: 10.3389/fcell.2021.711334 (PMC8453081; doi:10.3389/fcell.2021.711334)

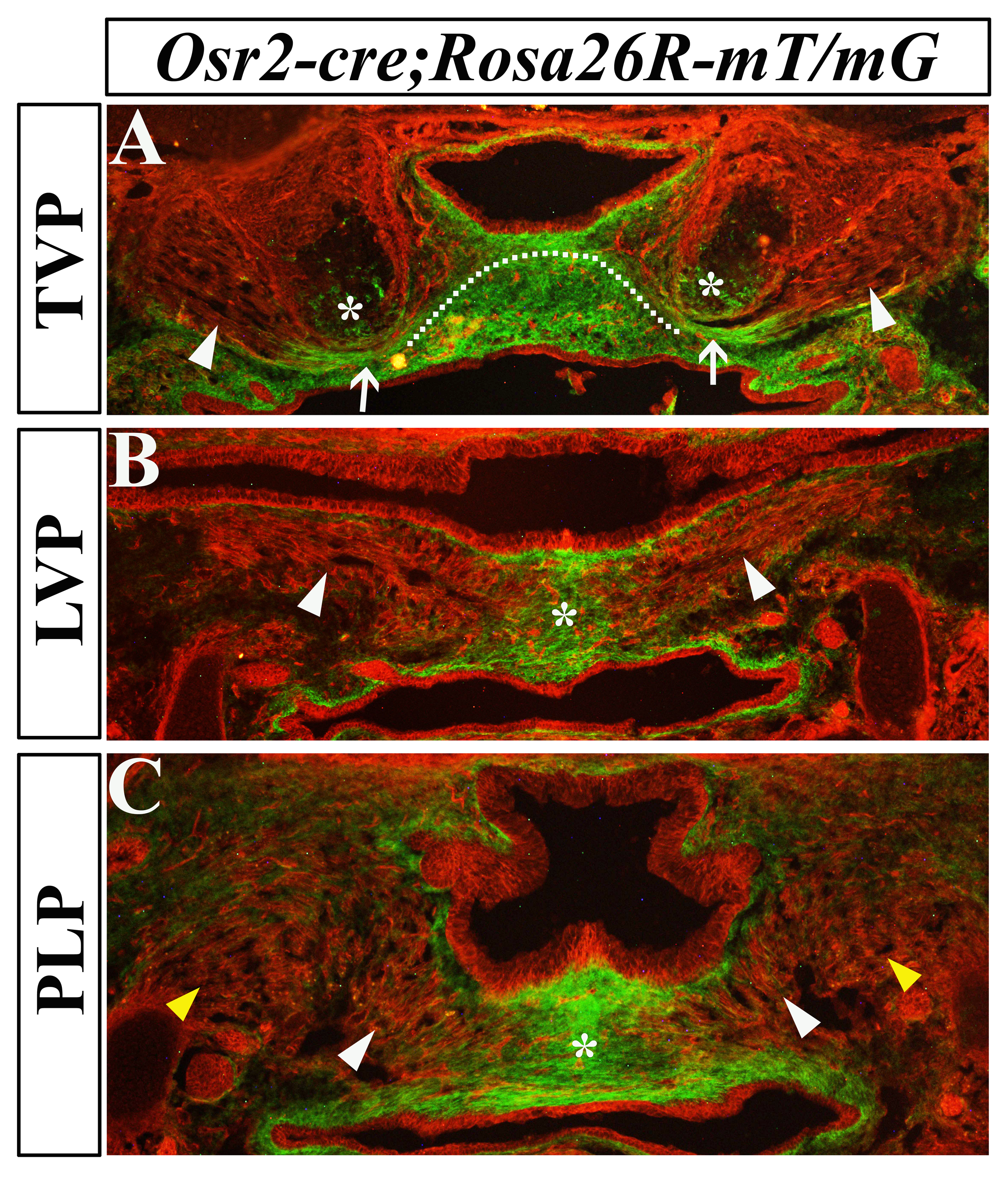

Supplement: Supplementary Figure 1 — The fluorescence pattern in Osr2-creKi; Rosa26R-mT/mG soft palate. (A) The Tvp level of soft palate in E16.5 Osr2-creKi; Rosa26R-mT/mG mouse. White dotted line delineated aponeurosis; asterisk meant Hpp; the white arrows pointed to the Tvp tendons; white arrowheads pointed to Tvps. (B) The Lvp level in E16.5 Osr2-creKi; Rosa26R-mT/mG soft palate. White arrowheads delineated Lvp myofibers; asterisk meant the aponeurosis. (C) The Plp level in E16.5 Osr2-creKi; Rosa26R-mT/mG soft palate. White arrowheads delineated Plp myofibers; yellow arrowheads pointed to Spc myofibers; asterisk meant the connective tissue. (Tvp, tensor veli palatini; Lvp, levator veli palatini; Plp, palatopharyngeus). [file Image_1.TIF]

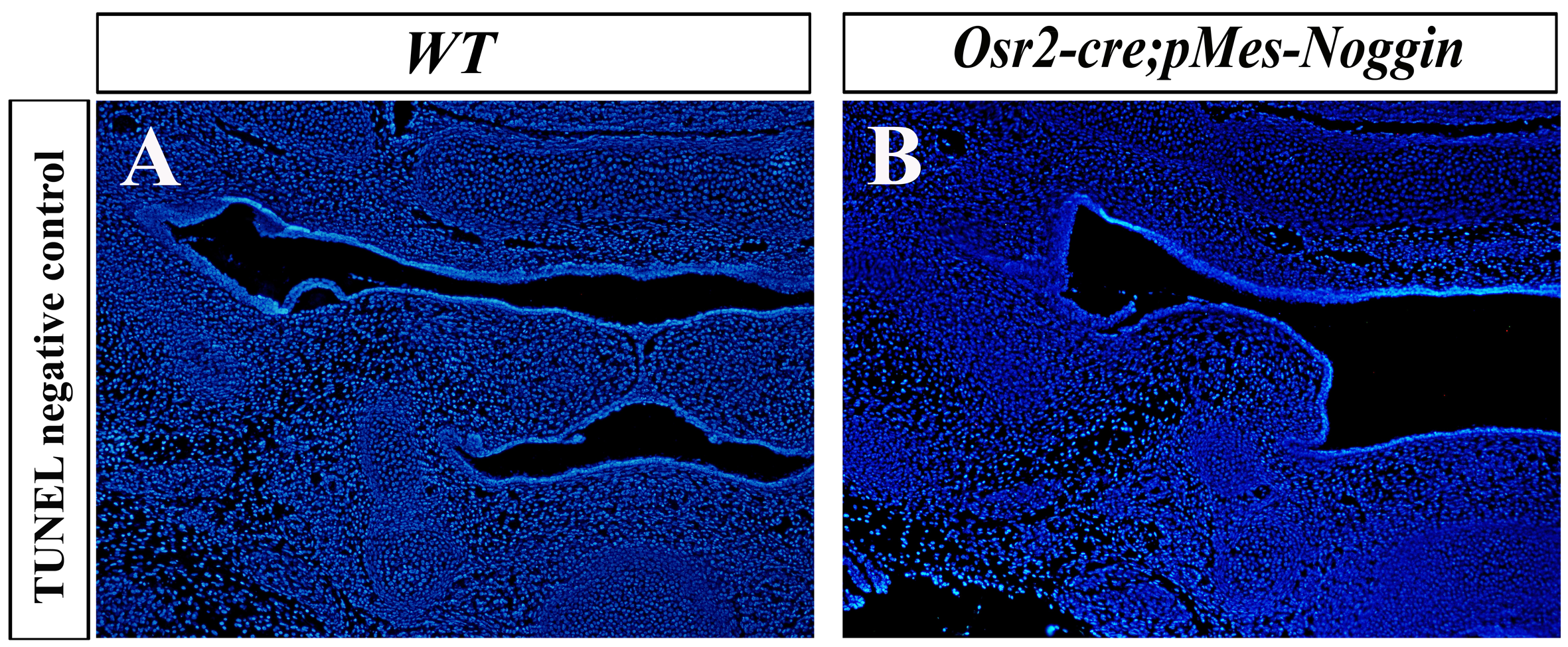

Supplement: Supplementary Figure 2 — The negative controls of Tunel assay. The Lvp levels in E14.5 Wt (A) and Osr2-creKi; pMes-Noggin soft palate (B). In both (A,B), the Tunel procedure followed the instruction in the In Situ Cell Death Detection Kit, except the solution of Blue Cap 1 was not added into the samples, but replaced by distilled water. So the TdT enzyme was left out, and no signal (green fluorescence) was detected. [file Image_2.TIF]
